# Comparative genomic analysis of *Lactobacillus plantarum* GB-LP4 and identification of evolutionarily divergent genes in high-osmolarity environment

**DOI:** 10.1007/s13258-017-0555-2

**Published:** 2017-11-16

**Authors:** Jaehoon Jung, Kwondo Kim, DongAhn Yoo, Chanho Lee, Jungsun Kang, Kyungjin Cho, Dae-Kyung Kang, Woori Kwak, Sook Hee Yoon, Hawsun Sohn, Heebal Kim, Seoae Cho

**Affiliations:** 1 Department of Agricultural Biotechnology and Research Institute of Population Genomics, Seoul National University, Seoul, 151-742 Republic of Korea; 2 C&K Genomics, 26 Beobwon-ro 9-gil Bldg. C, #1008 (H business park) Songpa-gu, Seoul, 05836 Republic of Korea; 3 Interdisciplinary Program in Bioinformatics, Seoul National University, Seoul, 151-742 Republic of Korea; 4 Genebiotech Co. Ltd., Seocho-Gu, Seoul, 137-787 Republic of Korea; 5 Department of Animal Resources Science, Dankook University, Cheonan, Republic of Korea; 6 Cetacean Research Institute, National Institute of Fisheries Science, Nam-gu, Ulsan, 44780 Republic of Korea

**Keywords:** *Lactobacillus plantarum*, Genome, Comparative genomics, Bacterial evolution, dN/dS

## Abstract

*Lactobacillus plantarum* is one of the widely-used probiotics and there have been a large number of advanced researches on the effectiveness of this species. However, the difference between previously reported *plantarum* strains, and the source of genomic variation among the strains were not clearly specified. In order to understand further on the molecular basis of *L. plantarum* on Korean traditional fermentation, we isolated the *L. plantarum* GB-LP4 from Korean fermented vegetable and conducted whole genome assembly. With comparative genomics approach, we identified the candidate genes that are expected to have undergone evolutionary acceleration. These genes have been reported to associate with the maintaining homeostasis, which are generally known to overcome instability in external environment including low pH or high osmotic pressure. Here, our results provide an evolutionary relationship between *L. plantarum* species and elucidate the candidate genes that play a pivotal role in evolutionary acceleration of GB-LP4 in high osmolarity environment. This study may provide guidance for further studies on *L. plantarum*.

**Electronic supplementary material:**

The online version of this article (doi:10.1007/s13258-017-0555-2) contains supplementary material, which is available to authorized users.

## Introduction

The major group of the lactic acid bacteria, *Lactobacillus* is commonly found in the human gastrointestinal-tract (De Vries et al. 2006), most of which inhabit in human body are widely known for their effectiveness. A number of previous studies also reported that *Lactobacillus* is showing anti-viral activities in animals and humans (Kechaou et al. 2013; Maeda et al. 2009; Park et al. 2013). Moreover, the characteristics of *Lactobacillus plantarum* strains that attract people’s attention include their antioxidant activities (Bested et al. 2013).

As one of the most well-known species of *lactobacillus, L. plantarum* is commonly found in fermented foods. Because many fermented foods are made through pickling process like sprinkling salts, bacteria which inhabit in fermented foods needs to be able to withstand extremely high osmolarity environment. While previous studies revealed that lactic acid bacteria have high survival rate on extreme environment (Chang et al. 2010; Conway et al. 1987; Shah and Jelen 1990), genetic causation of survival related to osmolarity or genomic differences between species have not been studied well in *plantarum* species.

In this study, we sequenced complete genome of *L. plantarum* GB-LP4, isolated from traditional Korean fermented vegetable. The whole genome assembly of the complete genome sequence of *L. plantarum* GB-LP4 was conducted which revealed the genomic contents of *L. plantarum*. Phylogenetic analysis revealed the evolutionary relationship between *L. plantarum* GB-LP4 and other previously reported *L. plantarum* strains. In addition, comparative analysis was performed with other 12 complete genome sequences of *L. plantarum* strains. From this, we identified evolutionarily accelerated genes that function as transferases. Also, we found the genome of GB-LP4 strains are 97.7% identical to the ZJ316 strain isolated from feces of a healthy infant. These results can expand our understanding on the environmental adaptation of *L. plantarum* and provide alternative ways to make use of the *L. plantarum* GB-LP4 derived from Korean fermented vegetable.

## Materials and methods

### Strain isolation and whole genome sequencing

Extraction of genomic DNA of GB-LP4 strain was proceded and it was purified using UltraClean Microbial DNA Isolation Kit (MoBio, Carlsbad, CA, USA) according to the manufacturer’s protocol. The concentration and purity of the isolated DNA was determined using NanoDrop spectrophotometer (Thermo Scientific, Wilmington, DE, USA). Approximately 5 μg of the extracted genomic DNA was sheared mechanically into 8–12 kb fragments using Hydroshear system (Digilab, Marlborough, MA, USA). SMRTbell libraries were prepared for SMRT sequencing with C4 chemistry on a PacBio RSα (Pacific Biosciences, Menlo Park, CA, USA). Purification of the libraries was conducted using 0.45× AMPure XP beads to remove short inserts sized under 1.5 kb. The size distribution of the sheared DNA template was characterized using an Agilent 12000 DNA kit (Applied Biosystems, Santa Clara, CA, USA). The sequencing primers were annealed to the templates at a final concentration of 5 nM template DNA, and DNA polymerase enzyme C4 was added according to the manufacturer’s recommendations for small-scale libraries. A DNA/Polymerase Binding Kit P6 (Pacific Biosciences) was used to load the enzyme template-complexes and libraries onto 75,000 zero-mode waveguides (ZMWs). The DNA sequencing reagent 2.0 kit (Pacific Biosciences) was used to sequence SMRT cells using a 120-min sequence capture protocol a long with a stage start to maximize the subread length with PacBio RSα.

### Genome assembly and annotation

Raw sequence data was filtered and assembled using SMRT portal system. Due to the high error rate of this pre-assembled data, repetitive remapping and polishing process was carried out by 100% consensus accuracy. A tool “Circlator” was used to circularize the genome (Hunt et al. 2015). We used RAST annotation system to annotate our genome (Aziz et al. 2008). For functional annotation of annotated genome, COG database (Galperin et al. 2015) was used and annotation map was generated using DNA Plotter (Carver et al. 2009).

### Comparative genomics analysis

A total of 12 complete genome sequences, and 11 draft genome sequences (which is scaffold level) of *L. plantarum* strains were downloaded from NCBI database (http://www.ncbi.nlm.nih.gov/genome/genomes/1108) for comparative analysis. Average nucleotide identity (ANI) value was calculated for these 24 strains using JSpecies v1.2.1 (Richter and Rosselló-Móra 2009). In order to identify further difference of *L. plantarum* GB-LP4 from other *plantarum* strains, we built Ortholog gene set for 13 complete genome using MESTORTHO method (Kim et al. 2008). Using PRANK (Löytynoja and Goldman 2008), multiple sequence alignment was run for each ortholog gene. Then, GBlocks (Talavera and Castresana 2007) was used to remove the poorly aligned sites. Finally, a total of 1681 orthologous gene sets were constructed and MEGA7 (Kumar et al. 2016) was used to build phylogenetic tree with neighbor joining method. In addition, bootstrap analysis was performed on the combined data set sequences. The maximum likelihood method (codeml of PAML4) (Yang 2007) was used to estimate the ratio of the rate of non-synonymous substitution to the rate of synonymous substitution, and evolutionarily accelerated genes based on branch and branch-site model. Visualization of multiple sequence alignment in evolutionarily accelerated gene from branch-site model was conducted using WebLogo3 (Crooks et al. 2004). Further comparative analysis with ZJ316 was conducted using Artemis comparison tool (Carver et al. 2005).

### Complete genome sequence accession number

The complete genome sequence of *L. plantarum* GB-LP4 have been deposited with the NCBI database under the accession number SUB1431878.

## Results

### General features of *L. plantarum* GB-LP4 genome

**Fig. 1:**
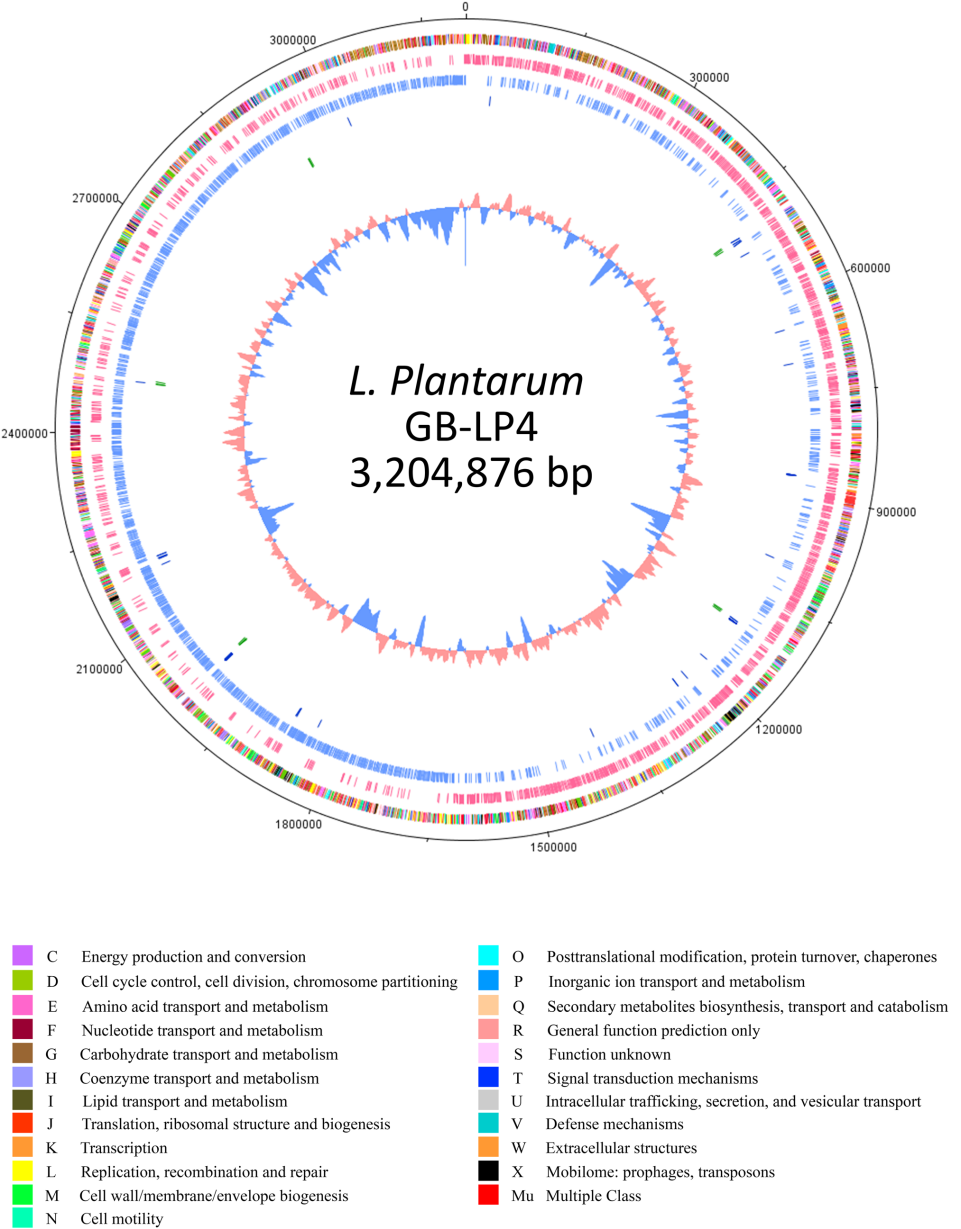
Genome map of *L. plantarum* GB-LP4. From *outer circle* to *inner circle*, each indicates the COG distributions, CDS in leading strand, CDS in lagging strand, tRNA, rRNA and GC contents ratio. Functional genes are labeled around the *outer circle* as follow

**Fig. 2:**
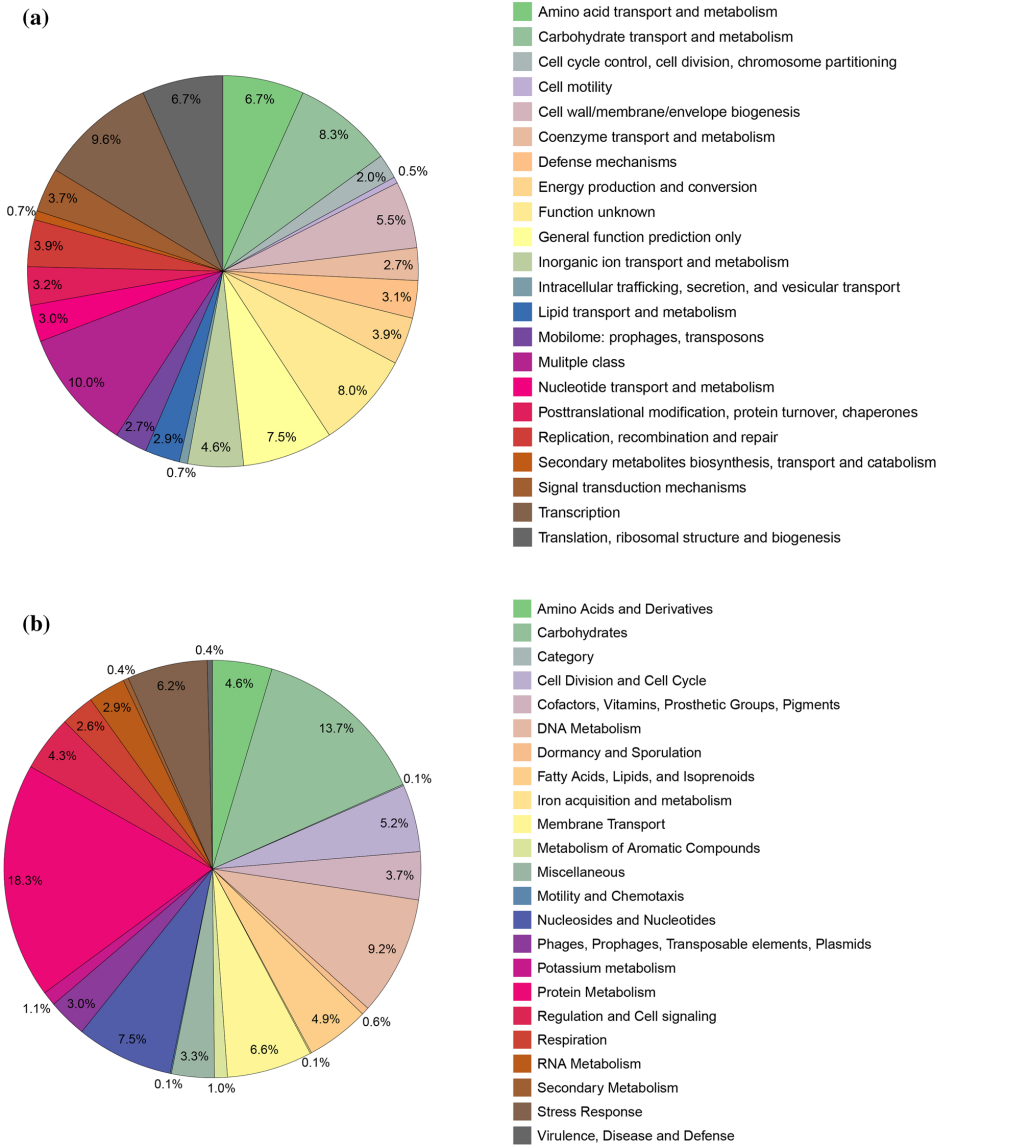
Functional categorization of all predicted ORFs in the genome of the strain GB-LP4 based on **a** COG and **b** SEED databases. The pie charts present that the proportion of functional categorization of ORFs in the genome based on two different databases

The genome of *L. plantarum* GB-LP4 was composed of a single circular DNA chromosome of 3,204,876 b.p. with 44.7% GC contents (Table 1). This genome contained 3,204 CDS (ORFs) and 85 RNAs (69 tRNA and 10 rRNA, 65S rRNA). Among the predicted ORFs, 756 genes (23.5%) were unknown or hypothetical genes (Fig. 1). The remainders of 2448 genes (76.5%) were expected to work as functional genes. Categorization of ORFs based on the SEED subsystem categorization and COG functional categorization are shown in Fig. 2.

**Table 1:** General features of *L. plantarum* strains

Strain	GB-LP4	ZJ316	P8	16
Genome size (b.p.)	3,204,876	3,203,964	3,035,719	3,044,678
GC contents (%)	44.7	44.6	44.5	44.7
Open reading frames	3204	3103	3080	3148
Functional genes	2448	2317	2216	2243
tRNA	69	61	68	66
rRNA	16	15	16	16
ANI (%)	100	99.94	99.15	99.23

Among 2448 genes, 1908 ORFs were categorized into 25 SEED subsystem categories. The subsystem “cofactors, vitamins, prosthetic groups, pigments” was assigned with 129 ORFs and 10 ORFs in this subsystem were related to Thiamin biosynthesis, which increases as the Korean traditional fermented vegetable ripens (Lee 1991). In addition, there were 49 ORFs, which were categorized into virulence, disease and defense. Among these ORFs, function of resistance to antibiotics and toxic compounds are most abundant (35 of 49). According to COG functional database, 2610 ORFs (81.4% of totally predicted ORFs) were assigned to COG functional categories. Among these, 1052 ORFs were included in the major five COG functional categories: 208 ORFs in category E (amino acid transport and metabolism), 255 ORFs in category G (carbohydrate transport and metabolism), 171 ORFs in category M (cell wall/membrane/envelope biogenesis), 298 ORFs in category K (transcription), and 120 ORFs in category J (translation, ribosomal structure and biogenesis).

### Comparative phylogenetic tree analysis

**Fig. 3:**
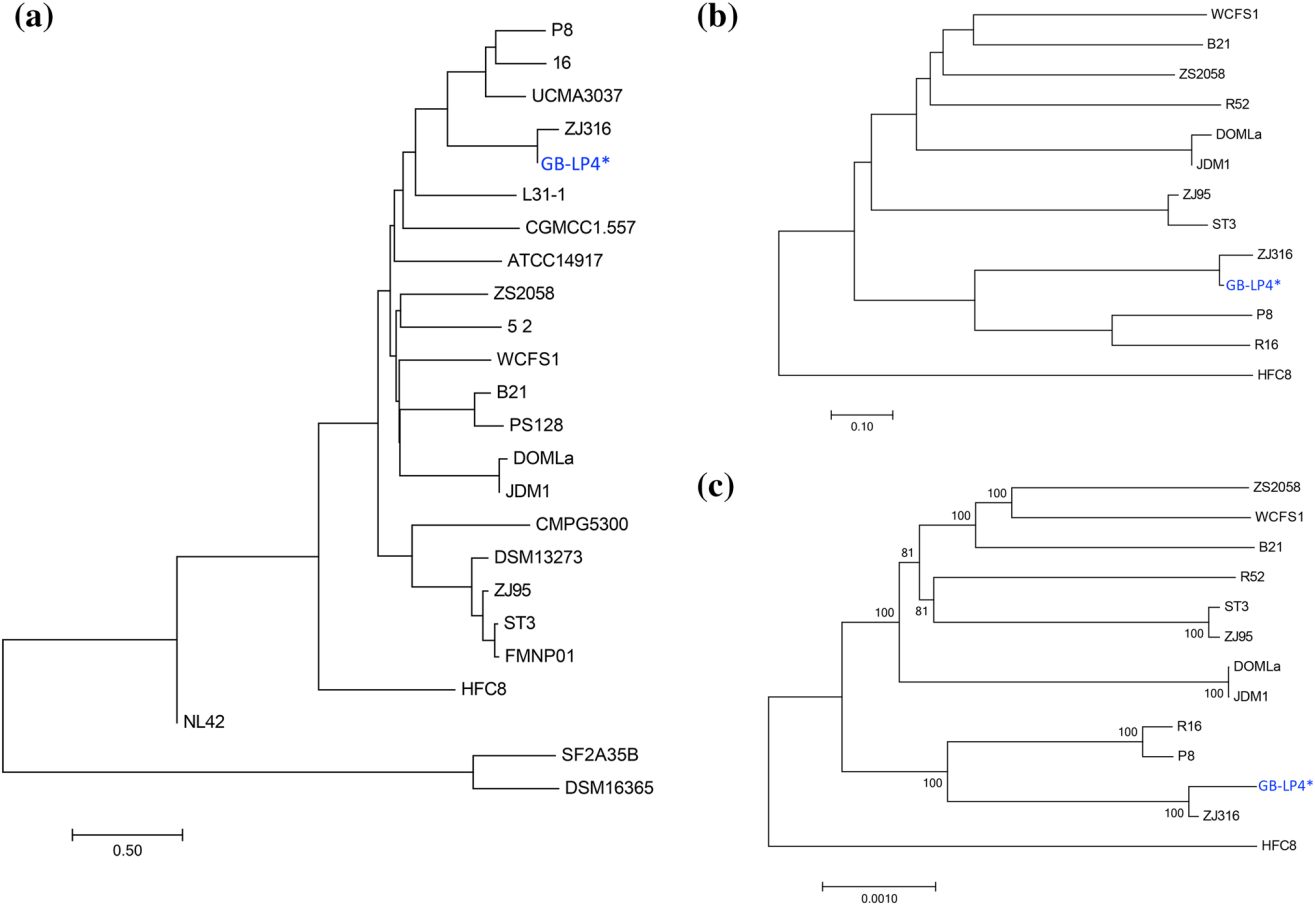
Comparative tree analysis. **a** ANI tree analysis of 24 genome sequences in *L. plantarum* strain using JSpecies. **b** ANI tree analysis of GB-LP4 with 12 available complete genome sequences of *L. plantarum*. **c** Phylogenetic tree analysis of GB-LP4 with seven available complete genome sequences using ortholog gene sets

For comparative tree analysis of GB-LP4 strain, two ANI trees and one phylogenetic tree were built. The two trees based on ANI values were constructed with 24 available genome sequences and 13 available complete genome sequences in NCBI database, respectively (Fig. 3). In every graph, four strains—P8, 16, UCMA3037, ZJ316-were grouped with GB-LP4 while ZJ316 being the closest strain to the GB-LP4 (99.94% of ANI value). The general features of these four strains are shown in the Table 1 for a comparison. ZJ316, which was isolated from feces of a healthy infant, was shown to have effects on pig growth and pork quality in previous study and it is predicted to inhibit of the growth of pathogens and promote increase in villus height. The phylogenetic tree was constructed using 1681 orthologous gene sets for 13 complete genome sequences. The tree was generated using neighbor joining method with bootstrap 1000 times (Fig. 3). This phylogenetic tree’s overall pattern was slightly different from ANI tree, and still the closest strain of GB-LP4 was ZJ316.

### Comparative genomics analysis

To reveal the evolutionary relationship between *strains*, we conducted dN/dS analysis using two models (branch, branch-site) and the results are shown in Table 2. In dN/dS analysis based on branch model, 2 genes [acetyltransferase (isoleucine patch superfamily), 2-C-methyl-d-erythritol 4-phosphate cytidylyltransferase] were identified as evolutionarily accelerated genes. In dN/dS analysis based on branch site model, only one gene was identified as evolutionarily accelerated gene. This gene functions as aspartate aminotransferase.

**Table 2:** Significant genes and relevant information in dN/dS analysis of branch model and branch-site model

Branch model			
Function	ω_F.G._	ω_B.G._	FDR
Acetyltransferase (isoleucine patch superfamily)	425.9473	0.0266	2.77E−06
2-C-methyl-d-erythritol 4-phosphate cytidylyltransferase	999	0.0363	2.06E−03

Branch-site model				
Function	Proportion of 2a	ω2_F.G._	ω2a_B.G._	FDR
Aspartate aminotransferase (EC 2.6.1.1)	0.0918	6.9596	0	2.91E−14

For further comparative genomic analysis, comparative genomic analysis of GB-LP4 and its closest strain ZJ316 was conducted. The result showed that nearly 97.7% (3,132,698/3,204,876 b.p.) sequences in GB-LP4 had 100% identity to ZJ316 and matching regions were shown in Fig. S1. We focused on the region that had relatively low identity (1,145,354–1,150,000 b.p., lower than 80% identity) and found that this region is known for the function of cardiolipin synthase in GB-LP4. Although the gene encoding cardiolipin synthase was found in other regions in the two strains, there are two genes encoding cardiolipin synthase in GB-LP4 whereas ZJ316 has only one gene encoding cardiolipin synthase.

## Discussion

The method of Korean-traditional fermentation involves the pickling process, which attenuates the number of microbes by hyperosmolarity. Nonetheless, GB-LP4 could survive in this high-osmolarity environment. Interestingly, our findings are closely associated with the GB-LP4’s adaptation to the high-stress environment. In branch model, a gene encoding 2-C-methyl-d-erythritol 4-phosphate cytidylyltransferase (EC 2.7.7.60) was identified to be positively selected. This enzyme catalyzes the formation of 4-diphosphocytidyl-2-C-methyl-d-erythritol from cytidine triphosphate and 2-C-methyl-d-erythritol 4-phosphate, and is involved in the non-mevalonate pathway of isoprenoid biosynthesis leading to the formation of isopentenyl pyrophosphate (IPP) and dimethylallyl pyrophosphate (DMAPP) (Kuzuyama and Seto 2012). In bacteria, Isoprenoid functions as cell wall biosynthesis intermediates. The gene most likely to have undergone positive selection in branch-site model was aspartate aminotransferase (EC 2.6.1.1). According to previous studies, decarboxylation of aspartate to alanine was found to take part in the generation of metabolic energy and regulation of intracellular pH in *Lactobacillus* (Abe et al. 1996; Konings et al. 1995). Concerning these observed properties, we speculated that aspartate aminotransferase might have a role related to maintaining homeostasis in high-osmolarity environment. Moreover, we have found that the gene encoding for cardiolipin synthase is located in the relatively low identity site between ZJ316 and GB-LP4. Osmotic induction of the gene encoding cardiolipin synthase contributes to the increasing proportion of cardiolipin as the proportion of phosphatidylethanolamine decreases in bacteria (Romantsov et al. 2009). Also, previous study found that the absence of cardiolipin synthase lead to significant uncoupling at the maximal rate of respiration and osmotic instability because of the strong interaction of cardiolipin with respiratory complexes (Koshkin and Greenberg 2002). From these findings, we speculated that the altered structure of evolutionarily selected genes in GB-LP4 would help to overcome instability in high osmolarity environment.

In conclusion, we performed comparative analysis on GB-LP4 and identified evolutionarily accelerated genes that are related to the survival of GB-LP4 in the specific environment—Korean traditional fermented vegetable. Further research on these genes will contribute to the understanding of adaptation of bacterial strains to a specific environment and the resulting phenotypic changes in bacteria.

## Electronic supplementary material

Below is the link to the electronic supplementary material.

Supplementary material 1 (PDF 22724 KB)
