# Supplementary material for: Comparative genomic analysis of Lactobacillus plantarum GB-LP4 and identification of evolutionarily divergent genes in high-osmolarity environment
Source: Genes Genomics. 2017 Nov 16;40(2):217–23. doi: 10.1007/s13258-017-0555-2 (PMC5846872; doi:10.1007/s13258-017-0555-2)
Supplement: Supplementary file 1 — Supplementary material 1 (PDF 22724 KB) [file 13258_2017_555_MOESM1_ESM.pdf]

(a)

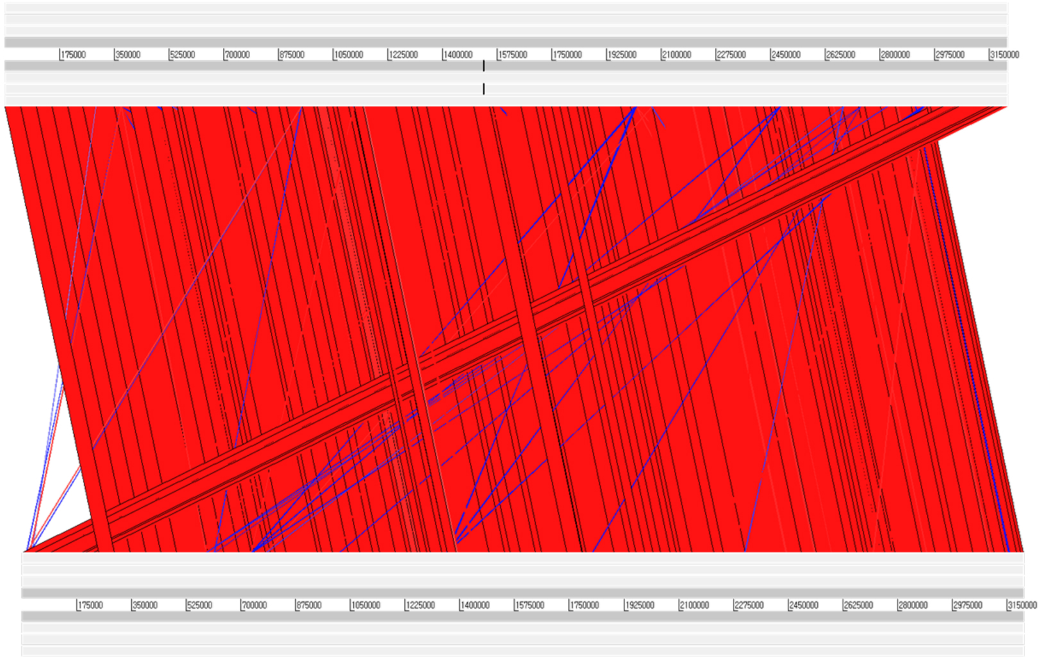

(b)

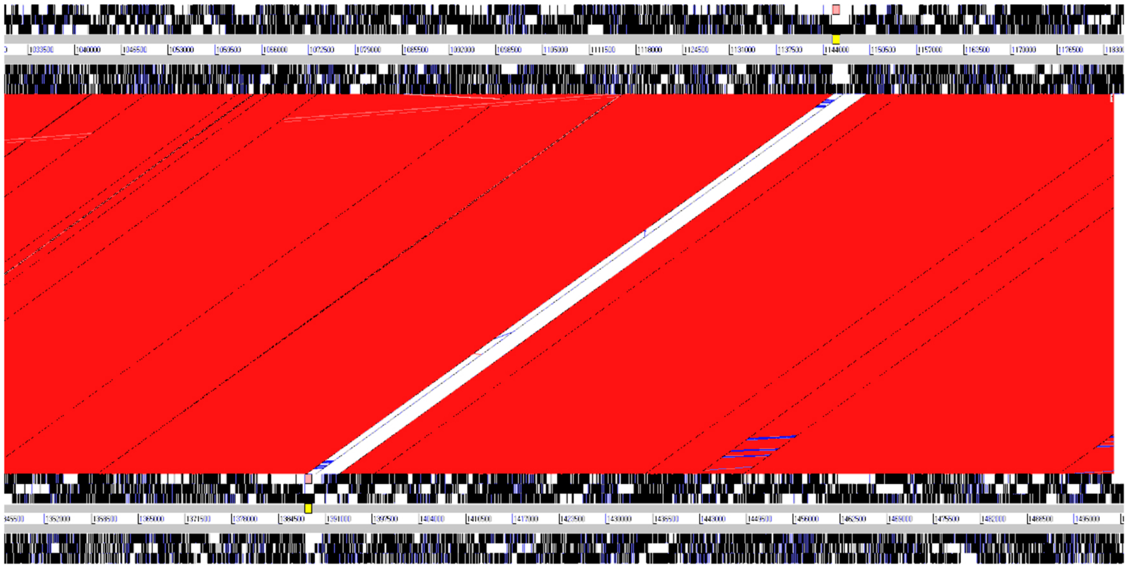

Supplementary Figure 1. Syntheny Block Analysis between LP4 (upper part) and ZJ316 (lower part) In whole genome (a), and in the region that has relatively low identity (b).
